# Risk Factors and Their Combined Effects on the Incidence Rate of Subarachnoid Hemorrhage – A Population-Based Cohort Study

**DOI:** 10.1371/journal.pone.0073760

**Published:** 2013-09-09

**Authors:** Miikka Korja, Karri Silventoinen, Tiina Laatikainen, Pekka Jousilahti, Veikko Salomaa, Juha Hernesniemi, Jaakko Kaprio

**Affiliations:** 1 Department of Neurosurgery, Helsinki University Central Hospital, Helsinki, Finland; 2 Neurosurgery Unit, Australian School of Advanced Medicine, Macquarie University, Sydney, New South Wales, Australia; 3 Population Research Unit, Department of Social Research, University of Helsinki, Helsinki, Finland; 4 Department of Chronic Disease Prevention, National Institute for Health and Welfare, Helsinki, Finland; 5 Institute of Public Health and Clinical Nutrition, University of Eastern Finland, Kuopio, Finland; 6 Hospital District of North Karelia, Joensuu, Finland; 7 Department of Public Health, University of Helsinki, Helsinki, Finland; 8 Department of Mental Health and Substance Abuse Services, National Institute for Health and Welfare, Helsinki, Finland; 9 Institute for Molecular Medicine FIMM, Helsinki, Finland; Charité University Medicine Berlin, Germany

## Abstract

**Background:**

Prospective studies on the risk factors for subarachnoid hemorrhage (SAH) are limited. Moreover, the effect of risk factors on the incidence rates of SAH is not well known about.

**Aims:**

In this study, we aimed to identify risk factors for SAH and characterize subgroups in a population with a high incidence of SAH.

**Methods:**

After recording multiple potential risk factors for SAH at the time of enrolment, first ever SAH events between 1972 and 2009 were recorded through the nationwide Causes of Death Register and Hospital Discharge Register for the population-based cohort of 64 349 participants, who participated in the National FINRISK Study between 1972 and 2007 in Finland.

**Results:**

During the follow-up time of 1.26 million person-years (median 17.9 years, range 0 to 37.9 years), 437 persons experienced fatal or non-fatal SAH. Crude SAH incidence was 34.8 (95% confidence interval: 31.7–38.2) per 100 000 person-years among ≥25-year-old persons. Female sex, high blood pressure values and current smoking were confirmed as risk factors for SAH. Previous myocardial infarction, history of premature stroke (any kind) in mother and elevated cholesterol levels in men were identified as new risk factors for SAH. Depending on the combination of risk factors, SAH incidence varied between 8 and 171 per 100 000 person-years.

**Conclusions:**

New and previously reported risk factors appear to have a much stronger association with the incidence of SAH than is ordinarily seen in cardiovascular diseases. Risk factor assessments may facilitate the identification of high-risk persons who should be the focus of preventive interventions.

## Introduction

The number of publications reporting risk factors for subarachnoid hemorrhage (SAH) are numerous, but only two large population-based prospective studies on SAH risk factors with a long (>10 years) follow-up time have been published to date [1], [2]. The results of these studies suggest that increasing age, female sex, hypertension and smoking are significant risk factors for SAH, whereas a high body mass index (BMI) may even decrease the risk of SAH [1], [2]. The results of many case-control studies are in line with these findings.

Using data from the ongoing national FINRISK Study [3], which was initiated in 1972 and is comprised of 5-yearly cross-sectional population surveys on risk factors of various diseases, we identified risk factors for SAH in a population-based cohort of nearly 65 000 Finnish citizens. Risk factor data was collected at enrolment, and a register-based follow-up was conducted from the enrolment to the onset of SAH, censoring, or the end of the follow-up period in December 2009. In addition, by performing risk factor profiling, we aimed to stratify subjects into subgroups with exceptional high incidence rates of SAH.

## Methods

### Ethics Statement

The approval for the FINRISK surveys has been applied each time from corresponding ethics committees. Informed consent (verbal until 1992 and written since 1997) was received from all participants.

### Data Collection

The data was drawn from the National FINRISK Study database [3]. The FINRISK surveys were initiated in 1972 and since then the surveys have been carried out every five years using independent, random and representative population samples from different geographical areas of Finland. Participants filled in a health-related baseline questionnaire and attended a clinical examination, which was conducted by trained research nurses at enrollment.

A register-based follow-up (based on the Hospital Discharge Register and the Causes of Death Register) was conducted for all participants from the enrolment to the FINRISK study to the onset of SAH (defined as first hospitalization for SAH or death from SAH if no hospitalization), as described previously [4]. In short, censoring occurred at the time of death or emigration (data from the Finnish Population Register), or at the end of the follow-up period in December 2009. The National Institute for Health and Welfare administers the Hospital Discharge Register, which includes the personal identification number, up to four discharge diagnoses (the first diagnosis being the principal cause of hospital stay), the dates of admission and discharge, and the hospital code. The Causes of Death Register is administered by Statistics Finland, where a specialist physician (forensic medicine) and a nosologist check the consistency of the underlying cause of death, and correct the diagnosis if necessary. Using the eight, ninth and tenth revisions of the International Classification of Diseases (ICD), incident cases of SAH were identified from the Hospital Discharge Register and from the Causes of Death Register. Data were retrieved on all persons listed with a diagnosis of SAH (ICD-8 and ICD-9 codes 430) or non-traumatic SAH (ICD-10 codes I60), and identified cases were linked to the baseline data using the personal identification number.

### SAH Study Cohort

Persons with a non-fatal SAH before the baseline (*i.e.* no prospective follow-up) were excluded. The SAH study cohort consisted of 64 349 Finnish residents, of which 33 235 (51.7%) were women. Mean age at enrolment was 45 years [standard deviation (SD) 12 years, range 25 to 74 years] with no difference by sex or future case status. In addition to the crude incidence of SAH, a European age-standardized incidence rate per 100,000 person-years was calculated using the European standard population to weight the age-specific rates.

### Analyzed Risk Factors

Analyzed risk factors included age, sex and questionnaire assessments (*i.e.* self-reporting) of smoking status, current alcohol consumption (data was available for cohorts examined between 1982 and 1997), previously diagnosed hypertension, previous stroke (any kind) (data was available for cohorts examined between 1982 and 2007), previous myocardial infarction (data was available for cohorts examined between 1982 and 2007), history of premature stroke [any kind of a stroke occurring before age 60 (cohort years 1972 to 1992) or 75 years (cohort years 2002 and 2007); age limit was not defined for the cohort year 1997] in father and mother, and history of premature myocardial infarction in father and mother [myocardial infarction/angina pectoris occurring before age 60 (cohort years 1972, 1977, 1982 and 1987) or myocardial infarction occurring before age 60 (cohort years 1992, 1997 and 2002) years; for the cohort year 2007, age limit for myocardial infarction in men and women was 60 and 65 years, respectively]. Current alcohol consumption was converted from Frequency and Quantity items as grams per week. Since more than 40% of the participants were non-regular users of alcohol, they could not be divided into two separate categories. Therefore, in order to divide alcohol consumption into fifths, we combined two fifths to create the non-users. Clinically measured risk factors included systolic and diastolic blood pressure (BP) (divided into fifths), BMI based on measured weight and height, and total serum cholesterol (divided into fifths).

### Statistical Analyses

We used the Cox proportional hazards model to estimate hazard ratios (HRs) of SAH with 95% confidence intervals. The time scale for the model was age (not time since entry), which was defined on the basis of birthdate, date at entry and date of exit from the study. In detail, the time scale is defined by birthdate but entry into the study was defined by the date of entry, and hence the follow-up time started from the age when entry took place. Exit took place at the time of diagnosis, censoring or end of follow-up. The use of this time scale adjusts for age. Departure from the proportional hazards assumption was evaluated by Schoenfeld residuals and by inspection of the “log-log” plots. Univariate analyses were adjusted for sex, and analyses were further stratified into two groups by sex. Multivariate analyses were adjusted for univariately identified risk factors, and also stratified by sex. Further adjustment by the survey cycle (or the study region) did not change results, and the survey cycle had no independent effect on the risk of SAH. Stratification by sex was used to illustrate possible modifications of HRs by sex, as also done previously [1]. Continuous risk factors were analyzed by categories based on quintiles. All analyses were performed using the Stata statistical software (Version 11.2 and 12.1, Stata Corp, College Station, Texas).

## Results

### Characteristics of the SAH Cases

Completeness of follow-up was 100% for deaths and hospitalizations. Only 0.4% of the FINRISK study participants are lost to follow-up due to emigration [5]. During the follow-up period of 1 256 535 person-years, 437 out of 64 349 persons had a fatal or non-fatal SAH (Table 1). The annual crude incidence of SAH was 34.8 per 100 000 person-years (≥25-year-old persons) (Table 1). The European standard population -adjusted incidence of SAH per 100,000 person-years was 19.0. Median follow-up time was 17.9 years (range 0 to 37.9 years) for all persons, and 12.3 years (range 0 to 37.7 years) for SAH cases. Median and mean age at diagnosis was 59.6 and 59.3 years (SD 12.3 years, range 26.0 to 91.2 years), respectively. Median and mean age at diagnosis was 57.4 and 57.2 years (SD 11.8 years, range 26.0 to 90.1 years), respectively, for men, and 61.8 and 61.0 years (SD 12.6 years, range 32.7 to 91.2 years), respectively, for women. 54.0% of the SAH cases were women (Table 1). Two-fifths (40.0%) died for SAH (any time) of all SAH subjects, and case fatality varied relatively little with age (Table 1). The prevalence of analyzed SAH risk factors is presented in Table 2.

**Table 1:** Person-years, number of cases, unadjusted SAH incidence per 100 000 person-years (95% confidence intervals [CI]), and case fatality of SAH by age group and sex.

Age (years)	Person years	SAH cases (women)	Incidence (CI)	Incidence inwomen (CI)	Incidence inmen (CI)	Case fatality %(women/men)
≤35	81 199	12 (3)	14.8 (8.4–26.0)	7.2 (2.3–22.4)	22.7 (11.8–43.7)	33.3 (66.7/22.2)
35.1–40	90 690	15 (8)	16.5 (10.0–27.4)	17.2 (8.6–34.5)	15.8 (7.5–33.1)	13.3 (12.5/14.3)
40.1–45	118 807	39 (23)	32.8 (24.0–44.9)	37.9 (25.2–57.0)	27.5 (16.9–45.0)	38.5 (39.1/37.5)
45.1–50	143 298	33 (16)	23.0 (16.4–32.4)	22.9 (13.4–35.8)	24.2 (15.0–38.9)	36.4 (43.8/29.4)
50.1–55	161 381	59 (23)	36.6 (28.3–47.2)	27.7 (18.4–41.6)	46.0 (33.2–63.8)	42.4 (34.8/47.2)
55.1–60	170 848	64 (33)	37.5 (29.3–47.9)	37.0 (26.3–52.0)	38.0 (26.7–54.0)	40.6 (36.4/45.2)
60.1–65	164 915	71 (36)	43.1 (34.1–54.3)	40.6 (29.3–56.3)	45.9 (33.0–63.9)	35.2 (33.3/37.1)
65.1–70	133 417	63 (38)	47.2 (36.9–60.4)	51.3 (37.3–70.5)	42.1 (28.5–62.3)	44.4 (36.8/56.0)
70.1–75	96 736	40 (26)	41.3 (30.3–56.4)	47.0 (32.0–69.0)	33.8 (20.0–57.1)	40.0 (38.5/42.9)
>75	85 242	41 (30)	43.0 (31.7–58.5)	49.8 (34.8–71.2)	31.5 (17.4–56.8)	53.7 (63.3/27.3)
All	1 256 535	437 (236)	34.8 (31.7–38.2)	35.1 (30.9–39.9)	34.4 (30.0–39.5)	40.0 (39.8/40.3)

**Table 2:** Prevalence of risk factors in the study cohort.

Risk factor (cases)	Subcategory	Overall %
Systolic BP (437)	≤122 mmHg	20.21%
	123–134 mmHg	23.36%
	135–144 mmHg	18.70%
	145–158 mmHg	19.28%
	≥159 mmHg	18.46%
Diastolic BP (437)	≤74 mmHg	22.40%
	75–82 mmHg	23.02%
	83–88 mmHg	18.57%
	89–94 mmHg	16.56%
	≥95 mmHg	19.45%
Diagnosed hypertension (428)		32.74%
Previous myocardial infarction (299)‡		2.51%
Previous stroke (296)‡		1.26%
Premature stroke in mother (416)		5.98%
Premature stroke in father (425)		5.97%
Myocardial infarction in mother (421)		11.74%
Myocardial infarction in father (425)		22.57%
Smoking (427)	Never	52.35%
	Quit >6 months ago	16.71%
	Quit <6 months ago	1.83%
	Occasionally	1.96%
	Daily (current)	27.15%
Alcohol consumption per week (171)‡‡	Non-user	42.19%
	1–36 grams	19.52%
	37–86 grams	18.34%
	≥87 grams	19.95%
Cholesterol levels (436)	≤4.92 mmol/l	20.08%
	4.93–5.58 mmol/l	20.02%
	5.59–6.22 mmol/l	19.91%
	6.23–7.06 mmol/l	20.00%
	≥7.07 mmol/l	19.99%
BMI (437)	18.5–24.9 kg/m^2^	41.82%
	<18.5 kg/m^2^	0.81%
	25–29.9 kg/m^2^	39.58%
	≥30.0 kg/m^2^	17.79%

‡ Data from the surveys available from 1977 to 2007, and ‡‡ from 1982 to 2007.

### Univariate Analyses of Risk Factors for SAH

#### Age and sex

SAH incidence increased especially between ages 45 and 70 years (Table 1). The incidence of SAH by 5-year age groups varied from 7.2 to 51.3 per 100 000 person-years (Table 1). The age-adjusted HR for women was 0.99 (95% confidence interval, 0.82 to 1.20) in comparison to men.

#### Blood pressure

Age and sex-adjusted HRs for SAH increased with systolic and diastolic BP values (measured at enrolment) (Table 3). For the highest systolic (≥159 mmHg) and diastolic (≥95 mmHg) BP fifths, the age and sex-adjusted HRs were 2.43 and 2.07, respectively (Table 3), in comparison to the lowest fifths of systolic (≤122 mmHg) and diastolic (≤74 mmHg) BP. Previously diagnosed hypertension increased the risk of SAH (Table 3).

**Table 3:** Major risk factors for SAH. Overall univariate analysis adjusted for age and sex.

Risk factor (cases)	Subcategory	Overall (CI)	Women (CI)	Men (CI)
Systolic BP (437)	≤122 mmHg	1	1	1
	123–134 mmHg	1.30 (0.91–1.86)	1.16 (0.73–1.83)	1.52 (0.83–2.80)
	135–144 mmHg	1.66† (1.17–2.38)	1.37 (0.85–2.21)	2.01* (1.12–3.63)
	145–158 mmHg	1.64† (1.14–2.33)	1.62* (1.03–2.55)	1.70 (0.93–3.10)
	≥159 mmHg	2.43† (1.72–3.41)	2.50† (1.64–3.83)	2.33† (1.28–4.24)
Diastolic BP (437)	≤74 mmHg	1	1	1
	75–82 mmHg	1.07 (0.74–1.53)	0.83 (0.52–1.32)	1.50 (0.83–2.71)
	83–88 mmHg	1.36 (0.96–1.95)	1.36 (0.87–2.12)	1.39 (0.77–2.54)
	89–94 mmHg	1.66† (1.18–2.35)	1.56* (1.01–2.43)	1.84* (1.04–3.27)
	≥95 mmHg	2.07† (1.49–2.88)	1.87† (1.23–2.86)	2.38† (1.38–4.12)
Diagnosed hypertension (428)		1.65† (1.36–2.00)	1.92† (1.48–2.49)	1.35* (1.00–1.82)
Previous myocardial infarction (299)‡		2.23† (1.27–3.93)	3.34† (1.46–7.60)	1.77 (0.82–3.81)
Previous stroke (296)‡		2.17 (0.97–4.90)	4.02† (1.64–9.86)	0.66 (0.09–4.74)
Premature stroke in mother (416)		1.57† (1.13–2.17)	1.50 (0.98–2.32)	1.65* (1.00–2.71)
Premature stroke in father (425)		1.23 (0.85–1.79)	0.98 (0.57–1.68)	1.56 (0.94–2.61)
Myocardial infarction in mother (421)		1.16 (0.86–1.55)	0.95 (0.63–1.43)	1.46 (0.97–2.20)
Myocardial infarction in father (425)		1.03 (0.81–1.30)	1.13 (0.83–1.55)	0.92 (0.64–1.31)
Smoking (427)	Never	1	1	1
	Quit >6 months ago	1.33 (0.96–1.84)	0.95 (0.51–1.76)	1.62* (1.03–2.56)
	Quit <6 months ago	1.82 (0.89–3.73)	1.85 (0.59–5.81)	1.91 (0.75–4.88)
	Occasionally	1.95* (1.06–3.59)	1.52 (0.62–3.73)	2.66* (1.12–6.35)
	Daily (current)	2.78† (2.21–3.50)	3.08† (2.30–4.11)	2.74† (1.87–4.03)
Alcohol consumption per week (171)‡‡	Non-user	1	1	1
	1–36 grams	1.05 (0.67–1.65)	0.88 (0.48–1.60)	1.40 (0.70–2.79)
	37–86 grams	1.42 (0.93–2.18)	1.17 (0.61–2.22)	1.69 (0.92–3.09)
	≥87 grams	1.95† (1.29–2.96)	3.14† (1.70–5.78)	1.66* (0.95–2.93)
Cholesterol levels (436)	≤4.92 mmol/l	1	1	1
	4.93–5.58 mmol/l	1.15 (0.79–1.69)	1.18 (0.74–1.88)	1.09 (0.56–2.12)
	5.59–6.22 mmol/l	1.38 (0.96–1.99)	1.05 (0.65–1.68)	1.97* (1.10–3.53)
	6.23–7.06 mmol/l	1.49* (1.05–2.12)	1.23 (0.78–1.94)	1.90* (1.07–3.40)
	≥7.07 mmol/l	1.55* (1.10–2.20)	1.13 (0.71–1.78)	2.26† (1.28–3.98)
BMI (437)	18.5–24.9 kg/m^2^	1	1	1
	<18.5 kg/m^2^	1.03 (0.33–3.22)	1.32 (0.42–4.18)	N/A
	25–29.9 kg/m^2^	1.04 (0.84–1.28)	1.06 (0.79–1.42)	1.00 (0.74–1.35)
	≥30.0 kg/m^2^	0.97 (0.82–1.20)	0.94 (0.65–1.35)	0.98 (0.63–1.52)

The results for women and men are derived from separate age-adjusted univariate analyses in women and men. Hazard ratios and 95% confidence intervals (CI). Total number of cases with information on the risk factor in parenthesis. BP = blood pressure.

* *P*<0.05,

† *P*<0.01,

‡ Data from the surveys available from 1977 to 2007, and ‡‡ from 1982 to 2007.

N/A, not applicable (too limited number of cases).

#### Smoking and alcohol consumption

Current smoking at enrolment increased the risk of SAH in comparison to never-smokers (Table 3). 82.7% of male and 39.8% of female SAH cases were ever-smokers at enrolment. 66.8% and 29.8% of all males and females in the cohort, respectively, were ever-smokers.

People using 87 grams or more pure alcohol per week (approximately 8 standard drinks per week) had a HR of 1.95 (Table 3) in comparison to abstainers. The HR was higher for women than men who used ≥87 grams alcohol per week (Table 3). Subgroup analyses by smoking status were not possible as only five out of 43 SAH cases, who reported increased alcohol use (≥87 g/week) at enrolment, were never-smokers.

#### Other potential risk factors

Previously experienced myocardial infarction was a predictor of a future SAH (Table 3). Premature stroke (any kind) in mother had a HR of 1.57 for SAH, whereas premature stroke (any kind) in father did not increase the risk of SAH significantly (Table 3). Cholesterol levels higher than 6.22 mmol/l increased the risk of SAH, especially among men (Table 3).

A history of prior stroke (any kind) had no significant association with a future SAH event (Table 3). Premature myocardial infarction in father or mother did not increase the risk of SAH (Table 2). BMI did not affect the risk of SAH (Table 3).

### Multivariate Analyses of Risk Factors for SAH

Due to the commonly known high correlation between systolic and diastolic BP values, only systolic BP values were selected for the multivariate analyses. All factors identified in the univariate analyses were included in multivariate analyses. Analyses were further stratified by sex, given the known differences in risk factor distributions between men and women. The first multivariate analysis indicated that alcohol consumption retained its significance (HR 1.83) as a SAH risk factor (Table 4). However, since data on alcohol consumption and previous myocardial infarction, too, were not available for all identified 437 SAH cases, the first multivariate analysis included only 145 cases. The second multivariate analysis, in which data on alcohol consumption and previous myocardial infarction were excluded, included 401 cases with all covariates (Table 5). Both first and second multivariate analyses showed that in addition to female sex, high (≥159 mmHg) systolic BP, previously diagnosed hypertension, history of premature stroke (any kind) in mother, elevated cholesterol levels in men, and current smoking remained statistically significant risk factors for SAH (Table 4 and 5).

**Table 4:** Multivariate analysis of the major risk factors for SAH.

Risk factor	Subcategory	Overall (CI)	Women (CI)	Men (CI)
Systolic BP	≤122 mmHg	1	1	1
	123–134 mmHg	1.97* (1.08–3.66)	1.61 (0.76–3.41)	3.31 (0.97–11.24)
	135–144 mmHg	2.07* (1.09–3.91)	1.17 (0.48–2.86)	4.07* (1.21–13.72)
	145–158 mmHg	1.78 (0.92–3.46)	1.86 (0.80–4.30)	2.31 (0.65–8.22)
	≥159 mmHg	2.64† (1.36–5.11)	2.75* (1.20–6.31)	3.44 (0.97–12.17)
Diagnosed hypertension		1.46* (1.02–2.09)	1.84* (1.09–3.09)	1.14 (0.68–1.90)
Previous myocardial infarction		3.07† (1.57–5.98)	4.50† (1.59–12.77)	2.60* (1.10–6.16)
Premature stroke in mother		2.08* (1.19–3.64)	2.37* (1.16–4.81)	1.71 (0.68–4.27)
Smoking	Never	1	1	1
	Quit >6 months ago	1.27 (0.76–2.15)	1.11 (0.46–2.68)	1.20 (0.60–2.42)
	Quit <6 months ago	2.54 (0.90–7.13)	3.77 (0.89–15.98)	1.75 (0.40–7.65)
	Occasionally	nc	nc	nc
	Daily (current)	3.10† (2.07–4.65)	3.92† (2.28–6.74)	2.28† (1.25–4.17)
Alcohol consumption/week	Non-user	1	1	1
	≤36 grams	1.30 (0.80–2.11)	0.99 (0.52–1.90)	1.85 (0.86–3.95)
	37–86 grams	1.58 (0.99–2.51)	1.17 (0.59–2.33)	2.01* (1.02–3.97)
	≥87 grams	1.83* (1.15–2.92)	2.30* (1.17–4.53)	1.75 (0.91–3.37)
Cholesterol levels	≤4.92 mmol/l	1	1	1
	4.93–5.58 mmol/l	0.94 (0.56–1.56)	1.02 (0.55–1.89)	0.80 (0.32–1.97)
	5.59–6.22 mmol/l6.23–7.06 mmol/l	0.98 (0.58–1.63)1.04 (0.62–1.75)	0.49 (0.22–1.08)0.78 (0.38–1.58)	1.82 (0.85–3.89)1.51 (0.68–3.35)
	≥7.07 mmol/l	0.95 (0.53–1.68)	0.51 (0.21–1.22)	1.73 (0.75–4.00)

Hazard ratios for all significant risk factors in the univariate analysis are presented, and the analysis includes FINRISK cohorts from 1982 to 2007 (Number of SAH cases with no missing covariate data = 145). There were no subjects who reported occasional smoking in this analysis (nc = no cases), as they were few in overall number in the study. Overall analysis adjusted for age, sex and all covariates in the table. The results for women and men are derived from separate multivariate analyses in women and men. 95% confidence intervals (CI).

* *P*<0.05,

† *P*<0.01.

**Table 5:** Multivariate analysis of the major risk factors for SAH.

Risk factor	Subcategory	Overall (CI)	Women (CI)	Men (CI)
Systolic BP	≤122 mmHg	1	1	1
	123–134 mmHg	1.26 (0.87–1.83)	1.18 (0.74–1.90)	1.37 (0.74–2.52)
	135–144 mmHg	1.51* (1.04–2.20)	1.37 (0.83–2.26)	1.64 (0.90–2.99)
	145–158 mmHg	1.43 (0.98–2.09)	1.56 (0.95–2.55)	1.33 (0.72–2.45)
	≥159 mmHg	1.89† (1.29–2.77)	2.00† (1.22–3.28)	1.81 (0.97–3.37)
Diagnosed hypertension		1.49† (1.20–1.85)	1.73† (1.29–2.33)	1.23 (0.88–1.71)
Premature stroke in mother		1.45* (1.04–2.03)	1.42 (0.92–2.19)	1.51 (0.91–2.53)
Smoking	Never	1	1	1
	Quit >6 months ago	1.37 (0.97–1.94)	1.02 (0.53–1.96)	1.42 (0.89–2.28)
	Quit <6 months ago	2.11* (1.03–4.33)	2.30 (0.73–7.27)	1.85 (0.72–4.74)
	Occasionally	2.33† (1.26–4.30)	1.89 (0.77–4.63)	2.68* (1.12–6.39)
	Daily (current)	3.10† (2.43–3.95)	3.67† (2.70–4.99)	2.53† (1.71–3.76)
Cholesterol levels	≤4.92 mmol/l	1	1	1
	4.93–5.58 mmol/l	1.02 (0.69–1.53)	0.99 (0.61–1.61)	1.09 (0.54–2.22)
	5.59–6.22 mmol/l	1.22 (0.84–1.78)	0.88 (0.54–1.44)	1.90* (1.02–3.56)
	6.23–7.06 mmol/l	1.28 (0.88–1.84)	0.98 (0.61–1.58)	1.85 (0.99–3.43)
	≥7.07 mmol/l	1.40 (0.97–2.01)	0.99 (0.62–1.59)	2.18* (1.19–4.00)

Hazard ratios for all other risk factors but alcohol and previous myocardial infarction are presented (Number of SAH cases with no missing covariate data = 401), and the analysis includes all FINRISK cohorts from 1972 to 2007. Overall analysis adjusted for age, sex and all covariates in the table. The results for women and men are derived from separate multivariate analyses in women and men. 95% confidence intervals (CI). *P<0.05, †*P*<0.01.

### Variation in SAH Incidence Depending on Risk Factors

**Figure 1:**
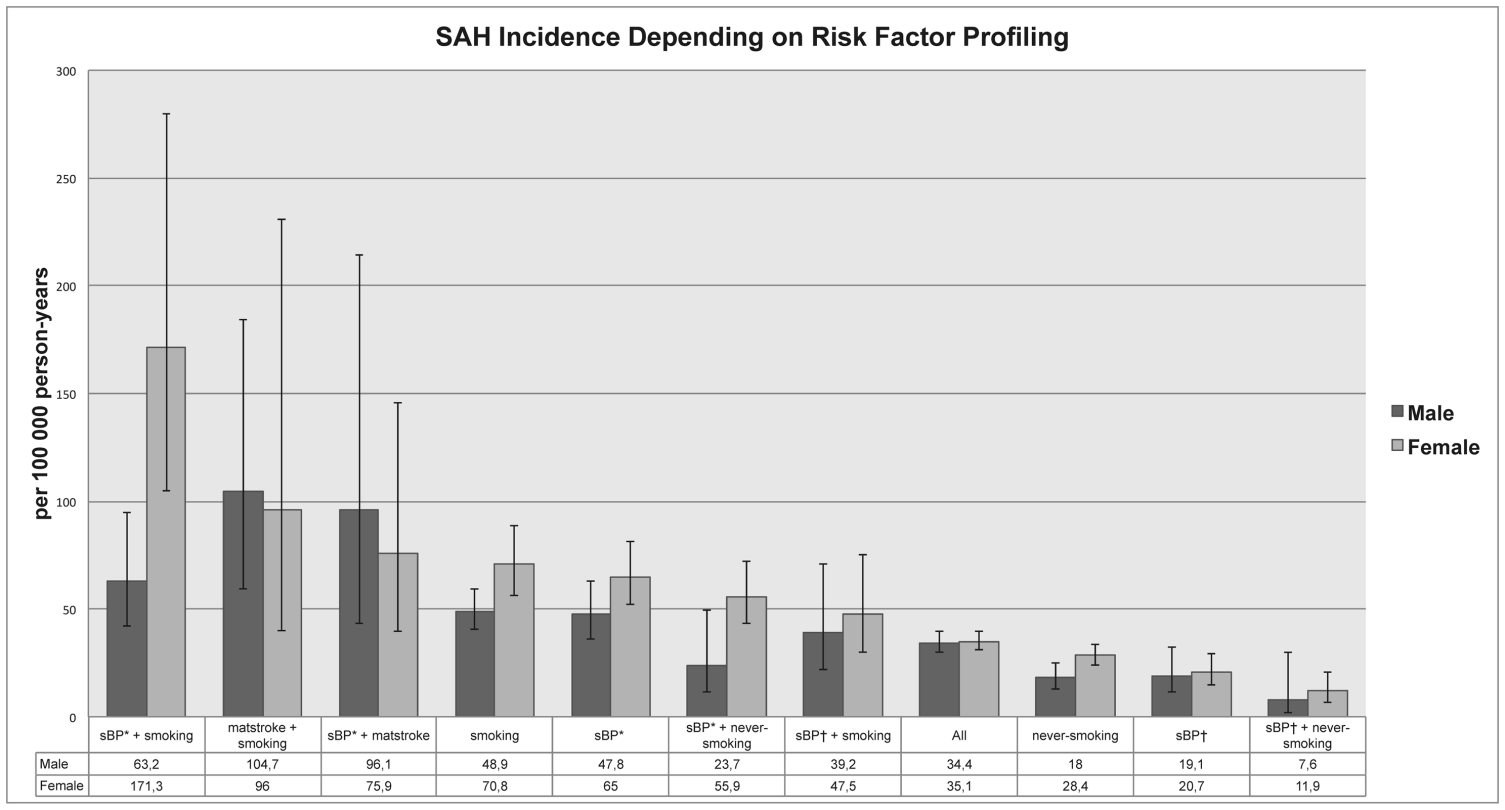
Crude SAH incidences in 25-year-old and older people on the basis of risk factor profiling. Error bars represent 95% confidence intervals. sBP* = systolic blood pressure ≥159 mmHg. sBP† = systolic blood pressure ≤122 mmHg. smoking = current smoking. matstroke = history of premature stroke (any kind) in mother.

We stratified the study sample by risk factor status and analyzed the incidence of SAH in various subgroups of the population-based cohort (Fig. 1). For example, never-smoking men with low systolic BP (≤122 mmHg) had SAH incidence of 8 per 100 000 person-years, whereas SAH incidence in smoking women with high systolic BP (≥159 mmHg) was 171 per 100 000 person-years (Fig. 1).

## Discussion

The presented results of this third large and long-term cohort study strengthen the previous [1], [2] prospective findings showing that female sex, high BP, and current smoking are major risk factors for SAH. Major risk factors appear to have a much stronger association with the incidence of SAH than is ordinarily seen in cardiovascular diseases, as the incidence of SAH varies substantially according to an individual risk factor profile. For example, smoking women with high systolic BP had 171 SAH cases per 100 000 person-years. Even though this study did not assess the risk of SAH in patients with unruptured intracranial aneurysms, these findings may be of assistance when estimating the risk of SAH in a patient with an unruptured aneurysm. Recent results from the prospective Norwegian study are in accordance with ours, and suggest that the risk of SAH increases exceptionally with a specific combination of risk factors [6]. Instead of calculating possible interactions using hazard ratio -based algorithms [6], we presented sex and risk factor -dependent incidence variations, which may be more applicable in clinical practice. National and cross-national differences for example in age, diagnostic accuracy of SAH, male-to-female ratio, smoking rates and prevalence of hypertension may partly explain the large variation in reported SAH incidences [7] between countries. The current results also suggest that previous myocardial infarction, elevated serum cholesterol levels especially in men and history of premature stroke (any kind) in mother are potential new risk factors for SAH. If these risk factors prove to be true in non-Finnish prospective studies, too, the etiology of SAH may need to be seen differently.

A recent large population-based cohort study suggests that smoking women have a higher risk of SAH than smoking men [8]. Interestingly, also the risk of coronary heart disease appears to be higher in smoking women than in smoking men [9]. Similar to the previous finding in the Mini-Finland study [1], the age-adjusted (not adjusted for other risk factors) risk of SAH was almost identical in women (HR 0.99 for women) and men. However, when adjusted for age and major risk factors in the multivariate analyses, female sex emerges as a significant baseline variable associated with an increased risk (HR 1.39–1.50) of SAH, as expected. Opposite to the findings of the previous prospective studies [1], [2], SAH incidence did not associate with baseline BMI. BMI values between current, former and never smokers may differ, and even if adjustment is made for current smoking status, changes in smoking habits may confound results suggesting an association between the SAH risk and BMI values.

The current study may have some advances in comparison with the two previous large cohort studies [1], [2]. First, we present a risk factor-based analysis of the variation in the incidence of a future SAH, which underlines the vast difference in the risk of SAH on the basis of risk factors. Such risk factor profiling has not been done in previous prospective studies. Second, the used National FINRISK Study cohort has the highest number of SAH events (n = 437), which, however, is still a limited number. Third, our study has the longest maximal follow-up time of 38 years. In general, our and previous [1], [2] cohort studies may provide more trustworthy evidence on SAH risk factors than numerous retrospective case-control studies, in part since collecting pre-morbid risk factor exposure data after a SAH event, especially when a fatal one, is often unreliable or even impossible in a retrospective study setup.

The current study has a number of limitations. First, the independent effect of increased alcohol use on the SAH risk could not be reliably determined, as it was difficult to tease apart the effect of smoking and alcohol, since only 12% of our SAH cases with high (≥87 grams alcohol/week) consumption were never-smokers. The multivariate analysis, in general, cannot fully adjust for all confounding factors, but does provide guidance on the relative importance of individual risk factors. Second, the study is a prospective one, but based on baseline pre-morbid data collection, not on longitudinal assessments. Therefore, no detailed exposure data for example of smoking was available, and thus single baseline risk factor measurements/assessments probably skew the value of assessed modifiable risk factors in predicting a future SAH event in this study. However, the data is still likely to be more reliable than in retrospective case-control studies, as already mentioned, and the previous large cohort studies [1], [2] are methodologically similar to ours. Third, in this study, the diagnosis of SAH was based on national registers, which are known to have errors. The accuracy of register-based diagnosis of SAH has been relatively high (79% and >90% accuracy for the Hospital Discharge Register and death certificates, respectively) at the era of the ICD-8 and ICD-9 diagnostic criteria [10], and when we assessed the data based only on ICD-10 diagnostic criteria (theoretically more reliable for diagnoses of aneurysmal SAHs), the results and conclusions remained practically the same (results not shown). Despite all this, the current study may include a small number of *e.g.* traumatic and non-aneurysmatic SAH cases, and thus overestimate the incidence of SAH and underestimate the impact of cardiovascular risk factors on SAH incidence and its variability. Fourth, the cohort size is still too limited to make more detailed risk factor profiling and subsequent risk analyses. Fifth, the study results may not be applicable to other than Finnish people. The incidence of SAH is commonly believed to be higher in Finland than elsewhere, even though the lack of national hospital and death registries (including autopsy reports for sudden deaths) in most of the world’s countries makes incidence comparisons difficult. The reported crude incidence of 34.8 per 100 000 person-years and the European standard population -adjusted incidence of 19.0 per 100 000 person-years among ≥25-year-old persons is comparable to the previously reported incidence of SAH in Finland, [1]. Sixth, the used stratification of BP and cholesterol levels into categories does not base on clinical cut-off values but quintiles. This may be acceptable as clinical cut-off values vary between countries and eras.

To conclude, female sex, high BP, and smoking are major risk factors for SAH, as already known previously, whereas previous myocardial infarction, elevated serum cholesterol levels especially in men and history of premature stroke (any kind) in mother may be new risk factors for SAH. These results suggest that SAH risk factors are somewhat similar to other cardiovascular diseases. The applicability of the presented risk factor profiling in identifying high-risk subjects of a future SAH may be worth studying in future, for example when planning screening and treatment strategies of unruptured intracranial aneurysms. Remarkable risk factor-dependent interindividual differences in the risk of SAH may have confounded the results and interpretations of some previous SAH studies.

### Presentation at a Meeting

Part of the study presented in the 63^rd^ Annual Meeting of the American Academy of Neurology, Hawaii, United States of America, April 9–16, 2011.
